# Editorial: Role of Sigma Factors of RNA Polymerase in Bacterial Physiology

**DOI:** 10.3389/fmicb.2021.633740

**Published:** 2021-03-26

**Authors:** Koichi Toyoda, Riccardo Manganelli, Miroslav Pátek

**Affiliations:** ^1^Research Institute of Innovative Technology for the Earth, Kyoto, Japan; ^2^Department of Molecular Medicine, University of Padua, Padua, Italy; ^3^Institute of Microbiology, Academy of Sciences of the Czech Republic (ASCR), Prague, Czechia

**Keywords:** sigma factor, RNA polymerase, stress response, anti-sigma factor, promoter, transcriptional start site

The transcription of bacterial genes driven by DNA-dependent RNA polymerase (RNAP) is the first step in gene expression. Holo RNAP consists of multi-subunit, catalytic core enzyme, formed by the α_2_ββ^′^ω subunits, and the sigma (σ) subunit, which confers the promoter selectivity of the RNAP. Bacteria usually contain several different σ factors to switch genes to be expressed. While the primary σ factor is responsible for housekeeping genes transcription and therefore essential, alternative σ factors are needed for the expression of genes induced in response to particular environmental stress or during development process. σ factors competitively bind to the core enzyme to form holo RNAP. To save cellular resources, they should be activated at an appropriate extent and timing. Therefore, the understanding of how the activity of σ factors is regulated and which genes are controlled by σ factors are important for understanding cellular responses to environmental changes.

This Research Topic contains three original research and three review articles. The half of the articles discuss how the activity of σ factors is regulated. The switching of a σ factor to form the holo RNAP is mainly regulated by the inhibitory interaction between σ factors and their cognate negative regulators, so-called anti-σ factors, which are modified and/or degraded in response to environmental stimuli. Guerreiro et al. reviewed the signal transduction cascade that activates SigB and the role of the SigB-mediated general stress response in the virulence of the pathogen *Listeria monocytogenes*. The multi-protein complex stressosome is an essential stress sensor, conserved in Firmicutes, which include *Bacillus subtilis* and *L. monocytogenes*. It is composed of the RsbS and RsbR paralogs and the serine/threonine kinase RsbT. RsbT is released from the complex upon sensing of stress and controls phosphorylation states of the anti-anti-σ factor RsbV, thereby releasing SigB from the anti-σ factor RsbW. The SigB regulon composed of more than 300 genes are important to resistance to stresses, including acid and osmotic stress, and bile salt, during infection of *L. monocytogenes*. Veyrier et al. show a novel functional aspect of an anti-σ factor, i.e., activating function. Natural mutations observed in the anti-σ factor RskA of the σ factor SigK in clinical isolates of mycobacteria conferred such an activity. Truncation of the C-terminal part identified the region required for the activator function of RskA. This activating function of the anti-σ factor was also demonstrated in different bacterial genera, indicating underappreciated functions of anti-σ factors. In addition to the step of the formation of holoenzyme, the step of transcription initiation is the target of transcription factors interacting with σ factors. Vishwakarma and Brodolin review such transcription factors regulating σ factors and the holo RNAP. In contrast to anti-σ factors, *Mycobacterium tuberculosis* RbpA and *Escherichia coli* Crl act as activators by stabilizing the interaction of a σ factor with the core enzyme and promoting the formation of open promoter complex for transcription initiation. They also review phage proteins repressing σ factor function of the host. The effects depend on promoter types.

The other three articles discuss the physiological function of σ factors. Extracytoplasmic function (ECF) σ factors comprise the largest group, Group 4, of the four groups in the σ^70^-family. Most of the members in this group are involved in the response to environmental stress. Temperature-dependent differential regulation of genes in an operon by two ECF σ factors in *Pseudomonas aeruginosa* was demonstrated by Bouffartigues et al. While heat shock induced all genes in the *cmaX*-*cfrX*-*cmpX* operon, encoding Mg^2+^-dependent channel, small protein of unknown-function, and mechanosensitive channel, respectively, by the σ factor AlgU, cold shock induced the latter two *cfrX*-*cmpX* from the SigX-dependent promoter located upstream of *cfrX* (intragenic region of *cmaX*). As the operon components encode proteins affecting membrane tension, which is influenced by cold shock, authors also showed that the drug valinomycin targeting the membrane induced SigX-mediated expression of the operon.

Transcriptional start site (TSS) determination is the first step to identify a promoter sequence. Recent high throughput sequencing technologies enable researchers to map the TSSs at the genome-wide level, identifying promoters of genes expressed under the growth conditions. Soutourina et al. determined 1,562 potential TSSs for *Clostridioides difficile* genes using 5′ end RNA-seq. σ factors were assigned based on comparison of the sequences preceding the TSSs with the consensus patterns of the promoters already identified in *C. difficile*. This analysis allowed refining the promoter consensus sequences. Moreover, in this topic, only this study treated a σ factor belonging to the σ^54^ family, represented by RpoN in *E. coli*. The σ^54^-type σ factors, including *C. difficile* SigL, recognize promoters consisting of −24 and −12 regions, not −35 and −10 regions as the σ^70^-type ones, with respect to the TSS, and require cognate transcriptional regulators for transcriptional activation. Another high throughput sequencing technology to identify promoter sequences is ChIP-seq. The complex of the target σ factor and DNA fragments encompassing its binding chromosomal regions is recovered by using the antibody specific to the σ factor. The recovered DNA fragments are amplified, sequenced, and mapped. RpoS orchestrates general stress response in *E. coli*, like SigB in *L. monocytogenes* described above. Schellhorn reviews the RpoS regulon, which is determined by transcriptome, proteome, and ChIP-seq, of *E. coli* strains. Based on the regulon components, RpoS functions as a metabolic switch. As described by Guerreiro et al., the regulons of the σ factors involved in general stress response are not only essential for metabolic adjustment and stress response but also represent burden for the growth, leading to the paradigm Self-Preservation and Nutritional Competence (SPANC). This may explain how, in many situations, mutations leading to RpoS loss of function can contribute to improved nutrient scavenging.

The articles in this special issue represent a part of roles of σ factors as molecular switches for physiological adaptation to intra/extracellular environmental changes. Although not specifically covered by this issue, recent structural studies revealed the molecular mechanisms of the “switches” in more detail (Glyde et al., 2018; Boyaci et al., 2019; Shi et al., 2020). We hope that this topic articles will contribute to presenting perspectives to authors in related fields and generally to researchers in molecular microbiology.

## Author Contributions

All authors listed have made a substantial, direct and intellectual contribution to the work, and approved it for publication.

## Conflict of Interest

The authors declare that the research was conducted in the absence of any commercial or financial relationships that could be construed as a potential conflict of interest.
